# Cytoplasm-predominant Pten associates with increased region-specific brain tyrosine hydroxylase and dopamine D2 receptors in mouse model with autistic traits

**DOI:** 10.1186/s13229-015-0056-6

**Published:** 2015-11-17

**Authors:** Xin He, Stetson Thacker, Todd Romigh, Qi Yu, Thomas W. Frazier, Charis Eng

**Affiliations:** Genomic Medicine Institute, Cleveland Clinic, 9500 Euclid Avenue, Mailstop NE-50, Cleveland, OH 44195 USA; Lerner Research Institute, Cleveland Clinic, Cleveland, OH USA; HHMI Graduate Program, Department of Molecular Medicine, Cleveland Clinic Lerner College of Medicine, Case Western Reserve University School of Medicine, Cleveland, OH USA; Center for Autism, Pediatrics Institute, Cleveland Clinic, Cleveland, OH USA; Department of Pediatrics, Case Western Reserve University School of Medicine, Cleveland, OH USA; Taussig Cancer Institute, Cleveland Clinic, Cleveland, OH USA; Stanley Shalom Zielony Institute of Nursing Excellence, Cleveland Clinic, Cleveland, OH USA; Department of Genetics and Genome Sciences, Case Western Reserve University School of Medicine, Cleveland, OH USA; CASE Comprehensive Cancer Center, Case Western Reserve University School of Medicine, Cleveland, OH USA

**Keywords:** PTEN, Autism, Gene, Dopamine, Tyrosine hydroxylase

## Abstract

**Background:**

Autism spectrum disorder (ASD) is a group of neurodevelopmental disorders characterized by impairment in social communication/interaction and inflexible/repetitive behavior. Several lines of evidence support genetic factors as a predominant cause of ASD. Among those autism susceptibility genes that have been identified, the *PTEN* tumor suppressor gene, initially identified as predisposing to Cowden heritable cancer syndrome, was found to be mutated in a subset of ASD patients with extreme macrocephaly. However, the ASD-relevant molecular mechanism mediating the effect of *PTEN* mutations remains elusive.

**Methods:**

We developed a *Pten* knock-in murine model to study the effects of *Pten* germline mutations, specifically altering subcellular localization, in ASD. Proteins were isolated from the hemispheres of the male littermates, and Western blots were performed to determine protein expression levels of tyrosine hydroxylase (TH). Immunohistochemical stains were carried out to validate the localization of TH and dopamine D2 receptors (D2R). PC12 cells ectopically expressing either wild-type or missense mutant PTEN were then compared for the differences in TH expression.

**Results:**

Mice carrying *Pten* mutations have high TH and D2R in the striatum and prefrontal cortex. They also have increased phosphorylation of cAMP response element-binding protein (CREB) and TH. Mechanistically, PTEN downregulates TH production in PC12 cells via inhibiting the phosphoinositide 3-kinase (PI3K)/CREB signaling pathway, while PTEN reduces TH phosphorylation via suppressing MAPK pathway. Unlike wild-type PTEN but similar to the mouse knock-in mutant Pten, three naturally occurring missense mutations of *PTEN* that we previously identified in ASD patients, H93R, F241S, and D252G, were not able to suppress TH when overexpressed in PC12 cells. In addition, two other *PTEN* missense mutations, C124S (pan phosphatase dead) and G129E (lipid phosphatase dead), failed to suppress TH when ectopically expressed in PC12 cells.

**Conclusions:**

Our data reveal a non-canonical PTEN-TH pathway in the brain that may work as a core regulator of dopamine signaling, which when dysfunctional is pathogenic in ASD.

**Electronic supplementary material:**

The online version of this article (doi:10.1186/s13229-015-0056-6) contains supplementary material, which is available to authorized users.

## Background

Autism spectrum disorder (ASD) is a group of neurodevelopmental disorders characterized by impairments in social interaction and communication, along with the presence of inflexible/repetitive behaviors [1]. Although a strong genetic component to ASD has been confirmed by behavioral genetic studies, only 10–20 % of cases have a known genetic etiology [2].

The phosphatase and tensin homologue deleted on chromosome ten (*PTEN*) gene encodes a dual-specificity phosphatase that suppresses the activity of the class Ia phosphoinositide 3-kinase (PI3K) pathway and the MAPK pathway [3–5]. Cowden syndrome, an inherited breast and thyroid cancer syndrome, is associated with germline mutations of the *PTEN* tumor suppressor gene on 10q23 [6, 7]. Subsequently, *PTEN* was shown to also play an important role in brain development and plasticity [8, 9]. In *Pten* conditional knockout mice, Pten deficiency in brain causes dramatically weakened synaptic transmission and defects in myelination of axons [10]. We first identified germline *PTEN* mutations in a subset of patients with ASD and extreme macrocephaly [11], an observation that was subsequently confirmed by multiple independent groups [12–14]. An estimated 7 % of children with ASD and macrocephaly carry a germline *PTEN* mutation [12]. Despite the macrocephaly, patients who have ASD and *PTEN* mutations have a cortex of normal thickness [15]. They also have an overgrowth of white matter and deficits in working memory and processing [15].

To provide insight into underlying causes for this disorder, we previously developed a germline *Pten* knock-in murine model of ASD. The male *Pten*^m3m4/m3m4^ mutant mice have extreme macrocephaly due to megencephaly [16]. They also mimic individuals with high-functioning ASD, displaying increased social motivation, poor balance, and normal recognition memory [17]. It is intriguing that germline mutations of *PTEN*, which predispose individuals to specific cancer types, can also be linked with ASD. Unfortunately, the molecular mechanism of *PTEN* mutation-positive ASD (herein, PTEN-ASD) remains unknown. Disturbed catecholamine metabolism has been reported in patients with ASD [18]. The urinary dopamine levels are reportedly significantly lower than those in normal children and are inversely correlated with the severity of autistic behavior [19]. Variants in the dopamine transporter gene (*DAT*) and the dopamine-3-receptor gene (*DRD3*) are associated with ASD [20, 21]. Adult mice with conditional Pten knockout in dopaminergic neurons exhibit elevated expression of tyrosine hydroxylase, the key enzyme for dopamine synthesis [22]. Based on the fact that *PTEN* deletion enhances survival and function of dopamine neurons [23], we sought to address the hypothesis that PTEN works as a key player in the regulation of the dopaminergic signaling in the brain, potentially through a non-canonical signaling pathway.

## Methods

### Murine model study

**Fig. 1:**
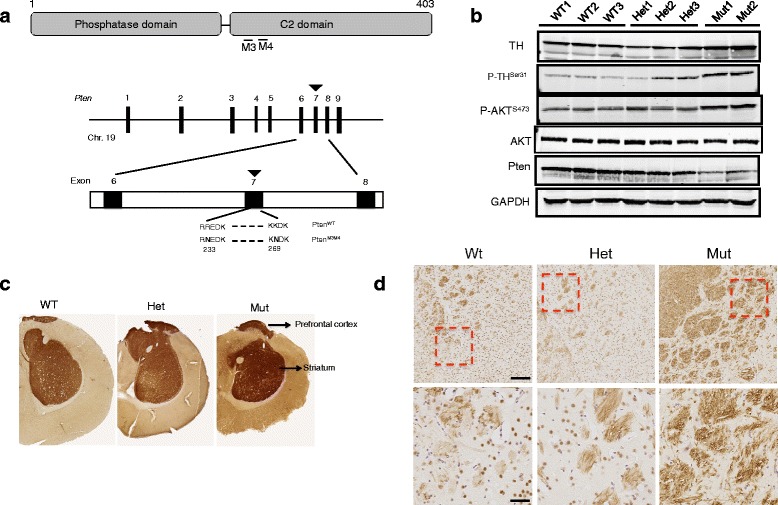
Pten mice have increased TH and P-CREB in the frontal cortex and striatum. **a** NLS-like region of *Pten* and the missense mutations (*Pten*, indicated in *bold*) created in the NLS-like region. **b** Western blotting results show elevated AKT and TH signaling, together with diminished Pten expression in the cerebral hemisphere of the Pten^m3m4/m3m4^ mutant mice. **c** Coronal section of the forebrain immunolabeled with the antibody against tyrosine hydroxylase (TH). Note the increased density of the TH-positive fibers in the striatum and frontal cortex of the Pten^m3m4/m3m4^ mutant mice. **d** Coronal section of the forebrain immunolabeled with the antibody against dopamine D2 receptors (D2R). Images were acquired at (*top*) ×5 and (*bottom*) ×20 magnification. Scale bar for *top panels* is 200 μm and *bottom panels* is 50 μm

*Pten*^m3m4^ missense knock-in mutant mice were generated in our lab and were backcrossed more than ten times onto a CD1 genetic background [24]. Animals were sacrificed at 8 weeks to harvest brains. The m3m4 dual *Pten* mutations are located in exon 7 (Fig. 1a). This exon is a hot spot for *PTEN* germline mutations in ASD patients (for instance, F241S, D252G). All protocols involving mice were approved by the Institutional Animal Care and Use Committee (IACUC) at the Cleveland Clinic.

### Reagents

Cycloheximide was purchased from Sigma-Aldrich (St. Louis, MO). The mitogen-activated protein kinase (MAPK)/ERK kinase (MEK) inhibitor, PD98059, was purchased from Calbiochem (La Jolla, CA). The PI3K inhibitor, LY294002, was purchased from Cayman Chemical (Ann Arbor, MI). The AKT inhibitor, Perifosine (>99 % pure), was purchased from LC laboratories (Woburn, MA). PTEN phosphatase inhibitor SF1670 was purchased from Cayman Chemical (Ann Arbor, MI). Nerve growth factor (NGF 2.5S) was purchased from Life Technologies (Grand Island, NY).

### Cell culture

The PC12 rat pheochromocytoma cell line was purchased from ATCC, where they authenticate by STRS analysis, and has endogenous wild-type *Pten*. We chose PC12 cells because it is one of the standard models for studying neural organs and, more importantly, to reflect both the ASD and neoplastic phenotypes seen in those with *PTEN* mutations. Mycoplasma contamination is routinely monitored. PC12 cells were grown in RPMI supplemented with 2 mM glutamine, 5 % fetal bovine serum (FBS), and 5 % horse serum. Experiments were performed on cells passaged no more than ten times.

### Generation of stable Tet-Off cell lines

The PC12 Tet-Off cell line was purchased from Clontech (Clontech Laboratories, Inc., Mountain View, CA, Cat#630906). Stable cell lines with Tet-regulated PTEN expression were generated as we described previously [25]. Tet-regulated expression of exogenous PTEN was confirmed by Western blot analysis on total protein lysates collected from cells grown in the presence or absence of Tet. The wild-type (WT) construct was used as the template to engineer each of the individual mutations (TetPTEN-H93R-FLAG, TetPTENF241S-FLAG, and TetPTEN-D252G-FLAG) to be tested. Each mutant was engineered using the QuickChange in vitro site-directed mutagenesis system (Stratagene) according to the manufacturer’s instructions.

### Cycloheximide chase study

PC12 cells stably expressing FLAG-tagged WT or mutant PTEN were incubated with cycloheximide (50 μg/mL) and were harvested at the indicated time points. Whole protein lysates were extracted and ran for Western blots using anti-FLAG antibody for transfected PTEN and GAPDH antibody as a loading control. Experiments were performed in triplicate.

### Plasmid electroporation

CREB Dominant-Negative Vector Set (including CREB, K-CREB, CREB133) was purchased from Clontech Laboratories (Mountain View, CA). For electroporation, 4D-Nucleofector was used according to the protocol from Lonza.

### Western blot

Whole cell lysates were extracted from brain tissues or PC12 cells by using RIPA buffer. Experiments were replicated in triplicate. All antibodies were purchased from Cell Signaling Technology (Beverly, MA) except tyrosine hydroxylase (TH) antibody, which was obtained from Abcam (Cambridge, MA).

### Immunohistochemistry

Briefly, mouse brains were fixed by intracardiac perfusion with 4 % paraformaldehyde in phosphate-buffered saline (PBS) and postfixation in the same buffer. Specimens were then embedded in paraffin, and sections processed for immunohistochemical staining with antibody against TH (1:100) or dopamine D2 receptor (1:50, Biorbyt Ltd, Cambridge, UK). Slides were scanned at ×20 using a Leica SCN400 slide scanner (Leica Microsystems Inc., Buffalo Grove, IL). Immunofluorescent staining was carried out with dopamine D2 receptor, and the slides were imaged at ×20 using a Leica DMI3000 B.

## Results

### *Pten*^m3m4^ mutant mice have increased TH in the prefrontal cortex and striatum

To address whether the TH and dopamine pathway in the brain plays an important role in ASD, we first examined the TH protein levels by Western blot in the cerebrum of male littermates aged 8 weeks (Fig. 1b). TH was significantly elevated in the mutant cerebrum (Fig. 1b). A similar trend of increase was also found for phospho-AKT^Ser473^ (P-AKT) and phospho-TH^Ser31^ (P-TH), whereas Pten protein was significantly diminished in the mutant mice when compared to the WT littermates (Fig. 1b).

To validate the region of elevated TH in the *Pten*^m3m4/m3m4^ mutant mouse brains, we then performed immunohistochemical staining of TH. Elevated TH was mainly distributed in the prefrontal cortex and striatum (Fig. 1c). Downstream of increased dopaminergic synthesis from TH is the dopamine D2 receptor (D2R), which plays critical roles in cognition. Therefore, we next measured brain D2R as a surrogate for the dopamine signaling pathway using immunohistochemistry. Consistent with the TH data, we found significantly increased D2R in the striatum and prefrontal cortex of the mutant mice (Fig. 1d). To further verify and quantify the increase in D2R expression in mutants, frontal sections of mouse brain were immunofluorescently stained, showing a significant increase in D2R expression in the mutant compared to WT (see Additional file 1: Figure S1). Taken together, these data suggest that the TH-dopamine-D2R pathway is upregulated in the striatum and prefrontal cortex of *Pten*^m3m4/m3m4^ mutant mice.

### PTEN suppresses TH expression via inhibition of the PI3K/CREB pathway

**Fig. 2:**
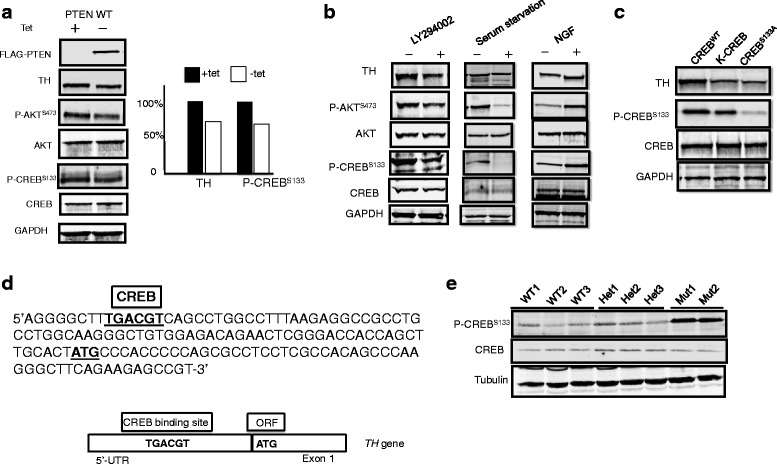
PTEN suppresses TH expression via inhibition of the PI3K/CREB pathway. **a** FLAG-tagged wild-type PTEN was expressed (Tet−, i.e., Tet-Off) or not expressed (Tet+) in PC12 cells, whose lysates were interrogated by Western blot for the labeled proteins/phosphoproteins (*left panel*). GAPDH is loading control. Note that FLAG-PTEN expression associates with decreased P-AKT (*right lane* vs *left lane*), decreased P-CREB, and decreased TH. Normalized quantitation of TH and P-CREB in the presence of PTEN overexpression (Tet−) or no PTEN overexpression (Tet+) (*right panel*). Note that Tet+ PC12 cells have endogenous rat wild-type Pten. **b** Western blots for named proteins/P-proteins after inhibition of PI3K by LY294002 (*left panel*) or serum starvation (*middle panel*) and after upregulation of PI3K by brief NGF exposure (*right panel*). Note that PI3K inhibition (manifested by increased P-AKT) is associated with decreased phospho-CREB and TH, whereas NGF stimulation of PI3K (manifested by decreased P-AKT) is associated with increased P-CREB and TH expression. **c** Western blot for TH, P-CREB, CREB, and GAPDH loading control after inhibition of CREB phosphorylation by expression of a CREB dominant-negative mutant (*K-CREB*) and a phosphorylation mutant (*CREB* ^*S133A*^) compared to wild-type CREB (*CREB* ^*WT*^). Note decreased TH expression associated with both (inactive) CREB mutants. **d** Identification of a CREB consensus binding site at the 5′UTR of the *TH* gene. **e** Elevated CREB phosphorylation in the cerebrum from *Pten* ^m3m4/m3m4^ mutant mice

The observation of increased AKT phosphorylation in the context of upregulation of the TH and dopamine pathways led to our hypothesis that this unexpected upregulation of the TH-dopamine pathway may be mediated by uncurbed PI3K signaling downstream of the *Pten* mutations. To test this hypothesis, we generated rat pheochromocytoma (PC12) cells that stably overexpress human PTEN under the control of the Tet-Off promoter, and the expression of TH was analyzed by Western blot. Ectopic PTEN expression was associated with decreased P-AKT and TH levels in PC12 cells (Fig. 2a, lane 2) compared to PC cells in its native state (Fig. 2a, lane 1). In this experiment, we were surprised to see that ectopic expression of human WT PTEN was associated with decreased phosphorylation of CREB (P-CREB) (Fig. 2a, right panel). Because CREB is a known regulatory target for AKT [26], we hypothesized that PI3K-dependent P-CREB allows TH transcription and hence translation.

To test our hypothesis that PI3K-dependent P-CREB facilitates TH transcription/translation, we sought to determine the levels of P-AKT, P-CREB, and TH after inhibition of the PI3K pathway as well as after stimulation of the PI3K pathway. If our hypothesis is correct, then inhibition of PI3K should result in the opposite of our above observation, i.e., decreased expression of TH, P-AKT, and P-CREB. We achieved PI3K inhibition via two different, and standard, approaches: pharmacologic inhibition of PI3K by LY294002 and serum starvation. First, we treated PC12 cells overnight with LY294002 (Fig. 2b, left panel, +). As a PI3K inhibitor, LY294002 was expected, and is observed, to decrease P-AKT expression (while AKT expression with and without treatment were identical). Notably, LY294002 also resulted in decreased TH and decreased P-CREB (CREB expression with and without treatment were similar) (Fig. 2b, left panel, + vs −). Similarly, serum starvation decreased P-AKT, as well as P-CREB, and TH (Fig. 2b, middle panel). We then chose NGF as our upregulator of PI3K and MAPK as an unrelated mimic of dysfunctioning/non-functioning PTEN: if our hypothesis remains correct, then NGF should be seen to increase P-AKT, P-CREB, and TH. When we exposed PC12 cells to NGF, Western blot indeed revealed increased expression of P-AKT, P-CREB, and TH (Fig. 2b, right panel). Thus, we were able to confirm that the PI3K pathway is important in mediating P-CREB, leading to transcriptional control of TH expression, at least in vitro.

To test whether CREB phosphorylation is sufficient to facilitate TH expression, we transfected PC12 cells with CREB^WT^ and two different CREB mutant constructs, CREB^S133A^ and K-CREB (Fig. 2c). CREB^S133A^ is a mutant in which serine 133, the major phosphorylation site of CREB, is changed to alanine, thus abrogating its ability to be phosphorylated. If our hypothesis is correct, then CREB^S133A^ should be associated with no/little P-CREB and decreased TH. Expression of the mutant CREB^S133A^ is, in fact, associated with dramatically decreased P-CREB, as expected; of note, CREB^S133A^ transfection is also associated with decreased TH (Fig. 2c, right lane), thus confirming our hypothesis. K-CREB is a dominant-negative mutant form of CREB. If our hypothesis is correct, K-CREB should decrease TH, without affecting P-CREB. Indeed, expression of K-CREB decreased TH without markedly reducing the P-CREB (Fig. 2c, middle lane). Of relevance, we identified, via bioinformatic analysis, a CREB consensus binding site at the 5′UTR of the TH gene (Fig. 2d). As partial in vivo validation of our observations in PC12 cells, we were able to show elevated CREB phosphorylation in the cerebrum of the *Pten*^m3m4/m3m4^ mice (Fig. 2e) where we have already shown increased TH expression (Fig. 1c). Taken together, our observations suggest that mutant PTEN is associated with upregulation of the PI3K pathway, manifested by increased P-AKT, resulting in CREB phosphorylation and subsequent increased TH transcription. Said another way, functioning (WT) PTEN suppresses TH expression via inhibition of the PI3K/CREB pathway.

### Wild-type PTEN reduces the phosphorylation of TH

We have already observed increased TH and increased phosphorylated TH in the cerebrum of our *Pten* mutant mice (above, Fig. 1b). Phosphorylation of TH is already known to increase TH protein stability and activity [27, 28]. Thus, we sought to determine if PTEN can also regulate TH phosphorylation.

**Fig. 3:**
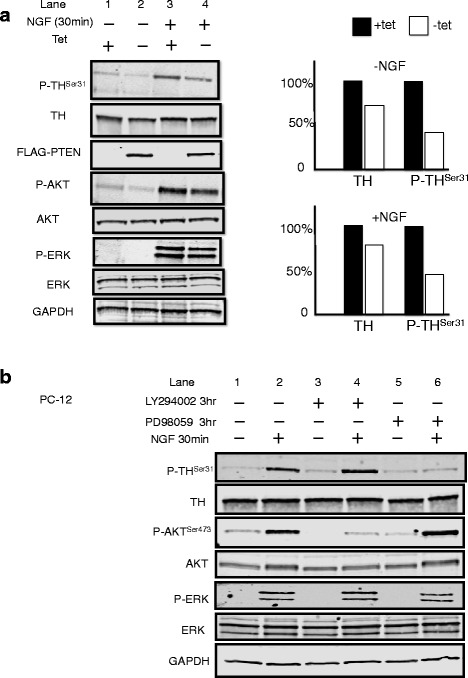
PTEN suppresses TH phosphorylation and stability via inhibition of the ERK/MAPK pathway in PC12 cells. **a** Western blot of the named proteins/phosphoproteins after exposure of NGF (to activate PC12 cells from resting state) or not, in the presence (Tet−) or absence (Tet+) of exogenous PTEN expression (*left panel*). Note that expression of PTEN reduces TH phosphorylation in PC12 cells. Normalized quantitation of relative TH and P-TH expression in the presence (*white bar*, Tet−) or absence (*black bar*, Tet+) of exogenous PTEN expression with (NGF+) or without (NGF−) exposure (*right bar graphs*). **b** Western blot of the named proteins/phosphoproteins in the presence (+) or absence (−) of MAPK inhibitor PD98059 or of PI3K inhibitor LY294002 with (+) or without (−) NGF stimulation of PC12 cells. Note that MAPK inhibitor PD98059, but not PI3K inhibitor LY294002, is associated with decreased P-TH even after NGF stimulation

PC12 cells at rest have low levels of P-TH (Fig. 3a, left panel, lane 1). We used NGF to make PC12 cells active; with only 30 min of NGF treatment, P-TH was significantly elevated (Fig. 3a, left panel, lane 3). The overexpression of PTEN (Tet−, i.e., Tet-Off turns on transfected PTEN) caused decreased P-TH (Fig. 3a, left panel, lane 4 [Tet−, PTEN on] vs lane 2 [Tet+, PTEN off]). After densitometry normalization, we found that total TH decreased ~20 % after exogenous PTEN expression, whereas the P-TH decreased ~50 % after exogenous PTEN expression (Fig. 3a, right panel). To further illustrate the effect of PTEN on TH phosphorylation, we used a PTEN inhibitor called SF1670 to inhibit both the ectopically expressed PTEN and the endogenously expressed PTEN in our PC12 cell line. A dramatic increase in phosphorylation at S31 on TH in PC12 cells was observed after treating for 3 h with the IC_50_ of SF1670. Moreover, a modest increase was observed in S40 phosphorylation. Consistent with our immunostaining of the m3m4 mouse brains, an increase in D2R expression was also observed after inhibition of PTEN with SF1670 (see Additional file 1: Figure S2). Therefore, these observations suggest that normal PTEN signaling inhibits TH expression (see above sections) as well as phosphorylation of TH.

PTEN can act as both a protein and lipid phosphatase that inhibits both MAPK and PI3K pathways. We have already shown (above sections) that PTEN inhibits the PI3K pathway to decrease CREB phosphorylation and total TH levels. Now, we would also like to determine if inhibition of MAPK and/or PI3K would affect TH phosphorylation. To minimize the effect of the change on total TH by chemical inhibitors, we only treated cells for 3 h with either the PI3K inhibitor, LY294002, or the MAPK inhibitor, PD98059, in media without serum. After 3 h, PC12 cells were treated with NGF for 30 min to awaken resting PC12 cells. NGF-stimulated PC12 cells had increased phosphorylation of TH without altering the level of total TH (Fig. 3b, lane 2). Surprisingly, PD98059, but not LY294002, inhibited NGF-stimulated TH phosphorylation in PC12 cells (Fig. 3b, lane 4 vs 6). Therefore, PTEN appears to suppress the phosphorylation of TH through inhibition of MAPK, probably through its protein phosphatase activity. Corroborating this, functional PTEN inhibitor SF1670, even in the presence of PTEN overexpression, resulted in increased P-ERK, slightly increased TH, and clearly increased P-TH (see Additional file 1: Figure S2 comprising lane 3 vs lane 2).

### Three PTEN missense mutants identified in ASD patients cannot suppress TH

**Fig. 4:**
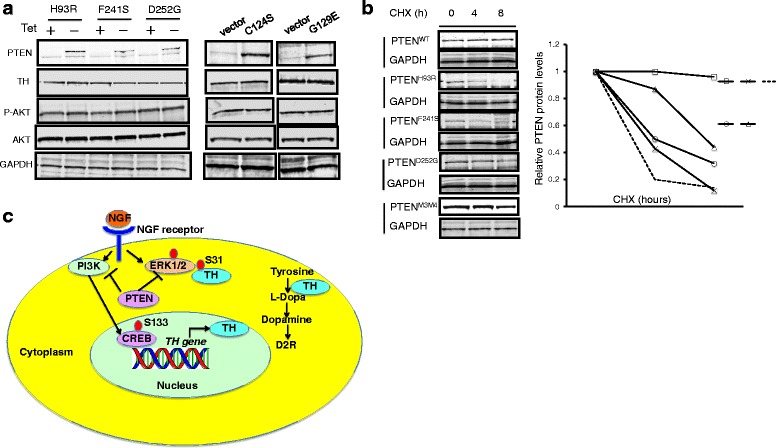
ASD-derived PTEN mutants fail to inhibit TH in vitro. **a** *Left panel*, loss of inhibitory effect of ASD-derived *PTEN* mutants on TH; PC12 cells stably expressed different PTENs were cultured in media with (Tet+) or without tetracycline (Tet−) [19]. TH was then measured by Western blot; *right panel*, tumor-associated *PTEN* mutants cannot inhibit TH. PC12 cells were transiently transfected with either vector or C124S and G129E mutant PTEN, and TH was measured by Western blot. **b** Cycloheximide chase reveals decreased protein stability of the ASD-derived PTEN mutants. **c** PTEN-induced molecular mechanism mediating the dopamine synthesis in the brain. PTEN suppresses NGF-induced activation of PI3K and ERK-MAPK pathways. PI3K phosphorylates CREB at Ser133 and further increases the transcription and translation of the *TH* gene. ERK-MAPK phosphorylates TH at Ser31 and increases the stability of the TH protein. Hyperactivity of both pathways caused by *PTEN* mutations leads to upregulated TH/dopamine synthesis and triggers the expression of the D2R receptors in neural tissue

In order to validate the role of *PTEN* mutations in ASD patients, we transfected three naturally occurring ASD-associated *PTEN* mutations into PC12 cells to see if these mutant PTEN constructs can suppress TH in vitro. If our mouse and PC12 data above are correct, then these three ASD-associated *PTEN* germline mutations should not be able to suppress TH levels. Indeed, all three mutations, H93R, F241S, and D252G, cannot suppress TH when expressed in PC12 cells. They also failed to suppress P-AKT (Fig. 4a, left panel). We then investigated TH expression after expression of *PTEN* mutations, C124S which is pan phosphatase dead and G129E which is only lipid phosphatase dead. Next, we investigated TH phosphorylation when these mutants were expressed in the presence of PTEN inhibitor SF1670. The three different *PTEN* mutations varied in their ability to suppress TH phosphorylation at S31 and S40; however, the general trend was an inability to suppress phosphorylation of TH as effectively as WT PTEN (see Additional file 1: Figure S3). Expression of both PTEN mutants in PC12 cells could not suppress P-AKT, TH, or P-TH (Fig. 4a, right panel). Again, our results underscore that the intact lipid phosphatase activity is necessary for PTEN to decrease TH expression, while intact protein phosphatase activity is necessary for PTEN to inhibit TH phosphorylation.

### Diminished protein stability in ASD-associated *PTEN* missense mutations

Based on our observations that the *Pten*^m3m4/m3m4^ mutant mice have diminished Pten protein levels in the brain [17], we hypothesized that ASD-associated *PTEN* mutations decrease the stability of PTEN protein. We therefore interrogated PTEN protein stability through a cycloheximide chase study (Fig. 4b). The WT PTEN protein was stable after synthesis and we observed that its levels remain at ~100 % even after 8 h of cycloheximide treatment. In contrast, H93R, F241S, and D252G and m3m4 PTEN/Pten mutant proteins are highly unstable. More than 50 % of the mutant m3m4 and D252G PTEN proteins were degraded after 8 h of cycloheximide treatment. For H93R and F241S, more than 80 % of the mutant PTEN protein was degraded after 8 h of cycloheximide treatment (Fig. 4b).

## Discussion

The present study demonstrates the increased expression and phosphorylation of TH in the prefrontal cortex and striatum of a *Pten* knock-in ASD mouse model. Our data indicate that functioning PTEN suppresses *TH* transcription through the inhibitory effect on PI3K and P-CREB, whereas PTEN also inhibits TH phosphorylation through suppressing the MAPK pathway (Fig. 4c). Thus, PTEN inhibits TH-dopamine pathway through its role as dual protein and lipid phosphatase.

Dopamine plays a key role in the regulation of locomotion, learning, behavior, emotion, and social interaction. TH is the rate-limiting enzyme in the synthesis of dopamine. Therefore, it is reasonable to posit that dysregulation of the dopaminergic system is linked to the pathophysiology of ASD. Of relevance, we have shown that mutations of the *PTEN* gene augment TH synthesis and function, and this may lead to the release of too much dopamine in the prefrontal cortex and midbrain, which can result in repetitive and obsessive behaviors. This can also explain the motor disorders (such as poor balance) reported in our mouse model [17]. Intriguingly, several groups have reported dysregulated dopamine function in ASD patients. Cartier et al. reported that two rare variants in *STX1A* and *SLC6A3* that associate with autism disrupt reverse transport of dopamine [29]. This same group also found that another variant of SLC6A3, the dopamine transporter gene, caused alterations in dopamine transport function and trafficking, which associated with ASD in two different families [30]. A Japanese group found that dopamine transporter binding was significantly higher in the orbitofrontal cortex of autistic individuals [31]. Furthermore, dysfunction of Pten has been associated with potential disruption of dopamine function and ASD behaviors in other mouse models. Conditional knockout of *Pten* in dopaminergic neurons resulted in neuronal hypertrophy, an increased number of dopaminergic neurons and fibers in the ventral mesencephalon, and social behavioral impairments [32, 33]. It has also been demonstrated that a germline *Pten* haploinsufficient model has altered social behavior, mirroring the social deficits of ASD, and displays neuronal overgrowth driven by β-catenin signaling [34, 35]. If the activated TH-dopamine pathway is germane to PTEN-ASD, there is a possibility that these symptoms could be reversed with pharmaceuticals that target the PTEN-TH-dopamine pathway.

The D2R represents the main autoreceptor of the dopaminergic system in the brain. The upregulation of striatal D2R expression is a notable finding in our study. A role for D2R in ASD susceptibility is suggested by the fact that risperidone, which prevents D2R activation, can reduce disruptive behavior in about half of the children with ASD [36]. In humans, D2R is encoded by the *DRD2* gene, which is a language-related gene. Variants in the *DRD2* gene, especially the rs1800498TT genotype and the A1 allele of the Taq I polymorphism, have been found to influence language traits in children with ASD [37–39]. Further studies of the effects of D2R on ASD will be required in order to assess whether common mechanisms are shared with what are reported herein for PTEN-mediated inhibitory effects on the dopamine pathway.

As a dual lipid and protein phosphatase, PTEN can suppress both the PI3K and the MAPK signaling pathways. We show that PTEN inhibits TH transcription by suppressing the PI3K and CREB pathways. We also show that PTEN can also suppress TH phosphorylation through its protein phosphatase activity by inhibiting ERK-MAPK. Thus, both lipid and protein phosphatase activities of PTEN are involved in TH regulation. The ASD-associated mutations of *PTEN* may present a “double whammy” in leading to ASD. Not only does PTEN signaling lessen total TH but it also reduces the phosphorylation of TH that leads to decreased activity and stability of TH protein. By using three ASD-associated *PTEN* missense mutations (H93R, F241S, D252G), we demonstrate the loss of PTEN’s inhibitory effects on P-AKT and TH and show that the lipid phosphatase activity of PTEN plays a major role in TH inhibition. Indeed, *TH* gene transcription is tightly controlled by the PTEN-PI3K pathway, as reported previously [40].

## Conclusions

Our observations reveal a novel inhibitory role of PTEN in the regulation of dopamine signaling via the non-canonical PTEN-TH-dopamine pathway in neural tissue. Mutations of the *PTEN* gene can enhance TH in two ways: the loss of the lipid phosphatase activity of PTEN will induce TH transcription and translation through the dysregulated PI3K/CREB pathway and the loss of the protein phosphatase activity of PTEN will enhance the phosphorylation of TH through the MAPK pathway. Continued investigation of the PTEN-TH-dopamine pathway in ASD will uncover novel drug targets for exploitation by new therapeutic interventions.

## Availability of supporting data

Supplementary information is available at Molecular Psychiatry’s website.

## Additional files

Additional file 1:
**Supplementary Information.** Three supplementary figures comprising **Figure S1A.** Immunofluorescent staining of frontal sections of mouse brains indicates increased D2R expression and larger nuclei in Pten^m3m4/m3m4^ mice. **Figure S1B.** Quantification of D2R immunofluorescence in triplicates of each genotype’s frontal section of mouse brains indicates the mutant D2R immunofluorescence is significantly increased in comparison to the immunofluorescence in the wild-type (*P* = 0.021). **Figure S2.** Inhibition of ectopic and endogenous PTEN function with SF1670 leads to an increase in phosphorylation of TH in PC12 cells. PTEN inhibitor is applied in lanes 1 and 3 (+) but off in lane 2 (−). Tetracycline is on (+) in lane 1, turning off ectopic expression of wild-type PTEN in PC12 cells that has endogenous wild-type PTEN. Tet-Off (−) turns on ectopic expression of wild-type human PTEN (lanes 2 and 3). **Figure S3.** Overexpression of naturally occurring ASD-associated germline *PTEN* mutations are unable to completely suppress TH phosphorylation. When tetracycline is applied (Tet+), ectopic PTEN expression is turned off. When tetracycline is removed (Tet-Off; −), ectopic expression of PTEN occurs. (PDF 965 kb)

## Abbreviations

ASD: autism spectrum disorder
CREB: cAMP response element-binding protein
D2R: dopamine D2 receptors
MAPK: mitogen-activated protein kinase
PHTS: PTEN hamartoma tumor syndrome
PI3K: phosphoinositide 3-kinase
TH: tyrosine hydroxylase
