# Erratum to: Cytoplasm-predominant Pten associates with increased region-specific brain tyrosine hydroxylase and dopamine D2 receptors in mouse model with autistic traits

**DOI:** 10.1186/s13229-016-0075-y

**Published:** 2016-02-03

**Authors:** Xin He, Stetson Thacker, Todd Romigh, Qi Yu, Thomas W. Frazier, Charis Eng

**Affiliations:** Genomic Medicine Institute, Cleveland Clinic, 9500 Euclid Avenue, Mailstop NE-50, Cleveland, OH 44195 USA; Lerner Research Institute, Cleveland Clinic, Cleveland, OH USA; HHMI Graduate Program, Department of Molecular Medicine, Cleveland Clinic Lerner College of Medicine, Cleveland Clinic Lerner College of Medicine, Case Western Reserve University School of Medicine, Cleveland, OH USA; Center for Autism, Pediatrics Institute, Cleveland Clinic, Cleveland, OH USA; Department of Pediatrics, Case Western Reserve University School of Medicine, Cleveland, OH USA; Taussig Cancer Institute, Cleveland Clinic, Cleveland, OH USA; Stanley Shalom Zielony Institute of Nursing Excellence, Cleveland Clinic, Cleveland, OH USA; Department of Genetics and Genome Sciences, Case Western Reserve University School of Medicine, Cleveland, OH USA; CASE Comprehensive Cancer Center, Case Western Reserve University School of Medicine, Cleveland, OH USA

## Erratum

We have just noticed a minor error in Fig. 1a of our article [1]. The m3m4 mutation was described incorrectly as it improperly describes two *Pten* mutations, R233N and K269N. However, the confirmed sequence data on the m3m4 mutation indicates there are five nucleotide changes, as we have previously published [2], resulting in four amino acid changes: R233Q, R234Q, K265N, and K266Q. The fifth nucleotide change is a synonymous mutation, L264L. For greater clarity on the details of the nucleotide changes and the corresponding amino acid changes of the m3m4 mutation, they have been provided:

**Fig. 1:**
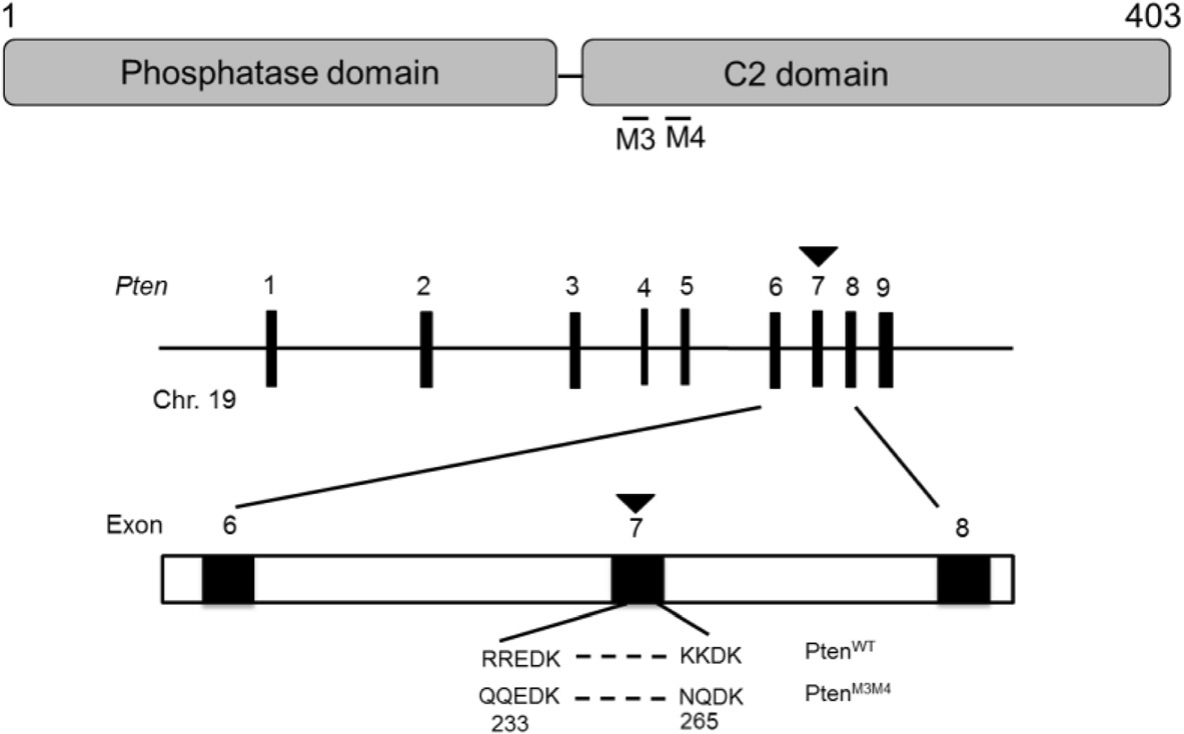
**a**. NLS-like region of *Pten* and the missense mutations created in the NLS-like region

Additionally, the included Fig. 1a now shows the correct amino acid changes. We apologize for any confusion caused by this error.

## References

[1] He X, Thacker S, Romigh T, Yu Q, Frazier TW, Eng C (2015). Cytoplasm-predominant Pten associates with increased region-specific brain tyrosine hydroxylase and dopamine D2 receptors in mouse model with autistic traits. Mol Autism.

[2] Tilot AK, Gaugler MK, Yu Q, Romigh T, Yu W, Miller RH, et al. Germline disruption of Pten localization causes enhanced sex-dependent social motivation and increased glial production. Hum Mol Genet. 2014;23(12): 3212–27. doi:10.1093/hmg/ddu031. 10.1093/hmg/ddu031 PMC4030776 24470394

