# Supplementary material for: Cytoplasm-predominant Pten associates with increased region-specific brain tyrosine hydroxylase and dopamine D2 receptors in mouse model with autistic traits
Source: Mol Autism. 2015 Nov 17;6:63. doi: 10.1186/s13229-015-0056-6 (PMC4647625; doi:10.1186/s13229-015-0056-6)
Supplement: Additional file 1: — Supplementary Information. Three supplementary figures comprising Figure S1A. Immunofluorescent staining of frontal sections of mouse brains indicates increased D2R expression and larger nuclei in Ptenm3m4/m3m4 mice. Figure S1B. Quantification of D2R immunofluorescence in triplicates of each genotype’s frontal section of mouse brains indicates the mutant D2R immunofluorescence is significantly increased in comparison to the immunofluorescence in the wild-type (P = 0.021). Figure S2. Inhibition of ectopic and endogenous PTEN function with SF1670 leads to an increase in phosphorylation of TH in PC12 cells. PTEN inhibitor is applied in lanes 1 and 3 (+) but off in lane 2 (−). Tetracycline is on (+) in lane 1, turning off ectopic expression of wild-type PTEN in PC12 cells that has endogenous wild-type PTEN. Tet-Off (−) turns on ectopic expression of wild-type human PTEN (lanes 2 and 3). Figure S3. Overexpression of naturally occurring ASD-associated germline PTEN mutations are unable to completely suppress TH phosphorylation. When tetracycline is applied (Tet+), ectopic PTEN expression is turned off. When tetracycline is removed (Tet-Off; −), ectopic expression of PTEN occurs. (PDF 965 kb) [file 13229_2015_56_MOESM1_ESM.pdf]

Supplementary Information

Cytoplasm-predominant Pten associates with increased region-specific brain tyrosine hydroxylase and dopamine D2 receptors in mouse model with autistic traits Pten

Xin He[1,2] Stetson Thacker[1,2,3] Todd Romigh[1,2] Qi Yu[1,2] Thomas W Frazier Jr[1,2,3,4,5] and Charis Eng[1,2,3,6,7,8,9]

[1]Genomic Medicine Institute, Cleveland Clinic, Cleveland, OH, USA [2]Lerner Research Institute, Cleveland Clinic, Cleveland, OH, USA [3]HHMI Graduate Program, Department of Molecular Medicine, Cleveland Clinic Lerner College of Medicine, Case Western Reserve University School of Medicine, Cleveland, OH, USA [4]Center for Autism, Pediatrics Institute, Cleveland Clinic, Cleveland, OH, USA [5]Department of Pediatrics, Case Western Reserve University School of Medicine, Cleveland, OH, USA [6]Taussig Cancer Institute, Cleveland Clinic, Cleveland, OH, USA [7]Stanley Shalom Zielony Institute of Nursing Excellence, Cleveland Clinic, Cleveland, OH, USA [8]Department of Genetics and Genome Sciences, Case Western Reserve University School of Medicine, Cleveland, OH, USA [9]CASE Comprehensive Cancer Center, Case Western Reserve University School of Medicine, Cleveland, OH, USA

Supplementary Figures

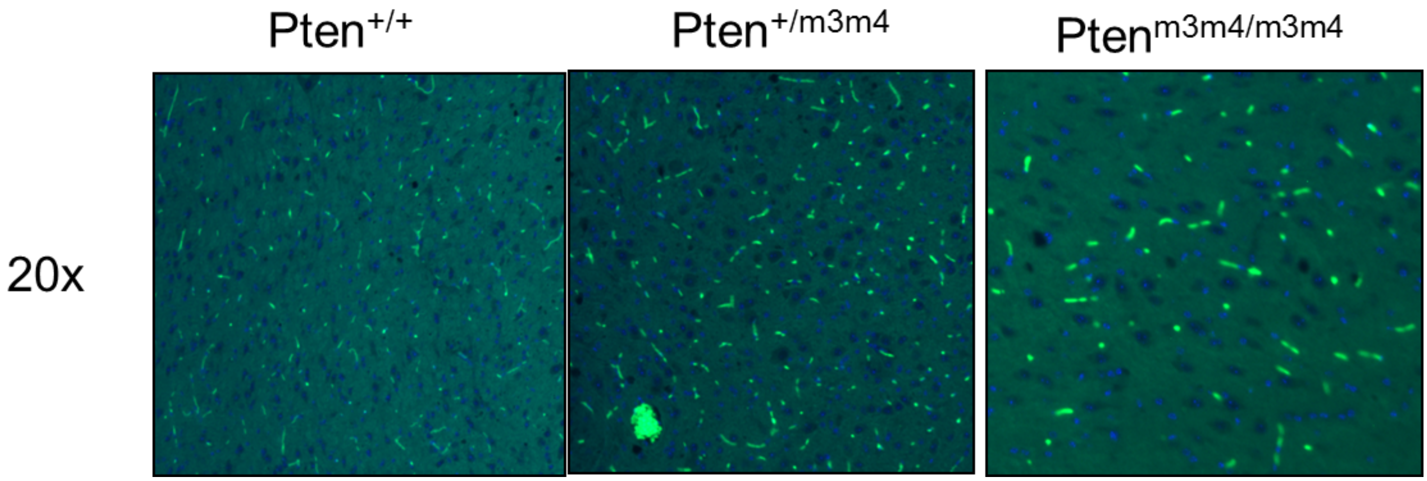

Supplementary Figure 1A. Immunofluorescent staining of frontal sections of mouse brains indicates increased D2R expression and larger nuclei in $Pten^{m3m4/m3m4}$ mice.

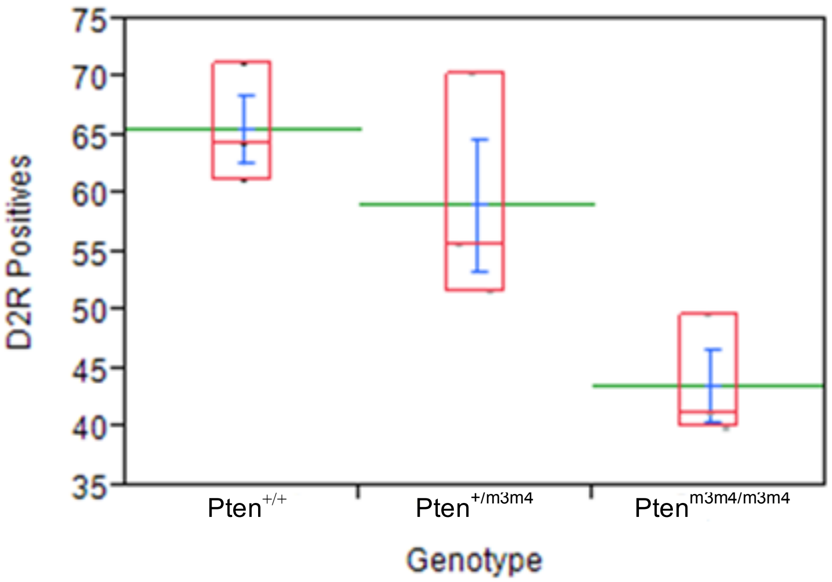

Supplementary Figure 1B. Quantification of D2R immunofluorescence in triplicates of each genotype's frontal section of mouse brains indicates the mutant D2R immunofluorescence is significantly increased in comparison to the immunofluorescence in the wild-type ($P = 0.021$).

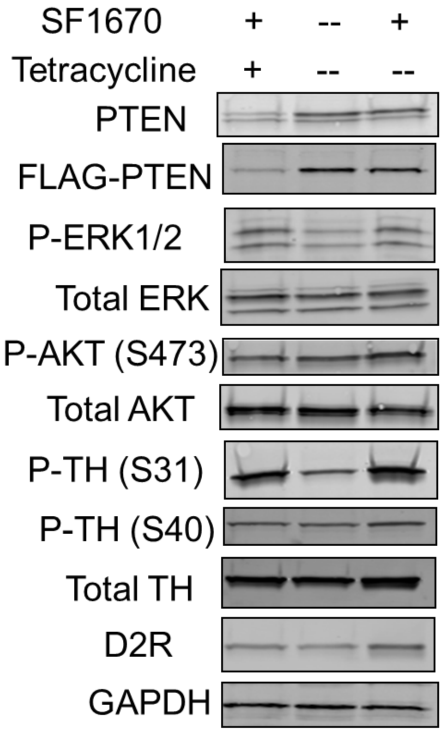

Supplementary Figure 2. Inhibition of ectopic and endogenous PTEN function with SF1670 leads to an increase in phosphorylation of TH in PC12 cells. PTEN inhibitor is applied in lanes 1 and 3 (+) but off (-) in lane 2. Tetracycline is on (+) in lane 1 turning off ectopic expression of wildtype PTEN in PC12 cells that has endogenous wildtype PTEN. Tet-off (-) turns on ectopic expression of wildtype human PTEN (lanes 2 and 3).

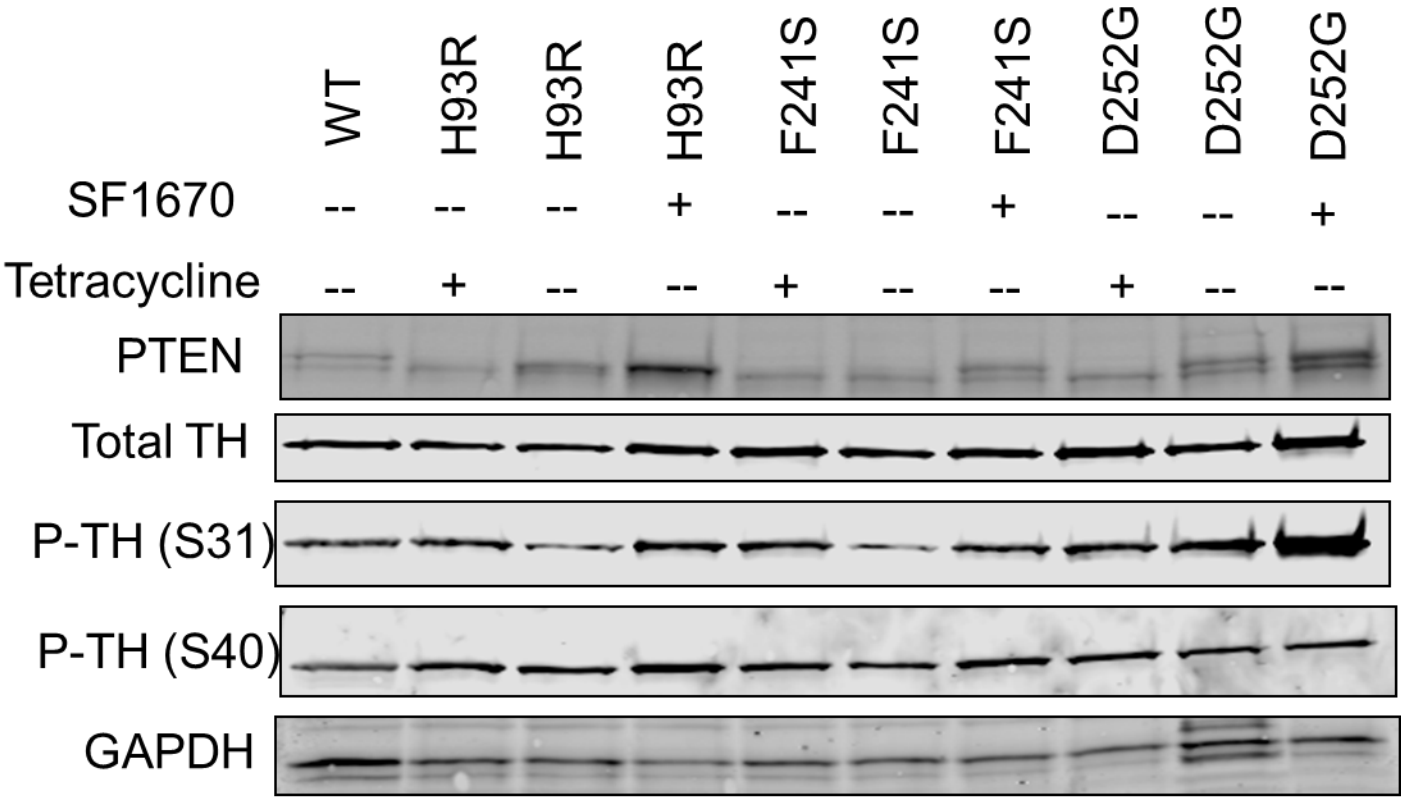

Supplementary Figure 3. Overexpression of naturally occurring ASD-associated germline *PTEN* mutations are unable to completely suppress TH phosphorylation. When tetracycline is applied (Tet+), ectopic PTEN expression is turned off. When tetracycline is removed (Tet-off; -), ectopic expression of PTEN occurs.
